# Effect of Austenitization Temperature on Hot Ductility of C-Mn-Al HSLA Steel

**DOI:** 10.3390/ma15030922

**Published:** 2022-01-25

**Authors:** Peter Prislupcak, Tibor Kvackaj, Jana Bidulska, Pavol Zahumensky, Viera Homolova, Lubos Juhar, Pavol Zubko, Peter Zimovcak, Roman Gburik, Ivo Demjan

**Affiliations:** 1 Research and Development USSE, U. S. Steel Kosice, s.r.o., Vstupny Areal U. S. Steel Kosice, 044 54 Kosice, Slovakia; pavolzahumensky@sk.uss.com (P.Z.); ljuhar@sk.uss.com (L.J.); pzubko@sk.uss.com (P.Z.); pzimovcak@sk.uss.com (P.Z.); rgburik@sk.uss.com (R.G.); 2 Department of Plastic Deformation and Simulation Processes, Institute of Materials and Quality Engineering, Faculty of Materials, Metallurgy and Recycling, Technical University of Kosice, Vysokoskolska 4, 042 00 Kosice, Slovakia; tibor.kvackaj@tuke.sk (T.K.); jana.bidulska@tuke.sk (J.B.); 3 Institute of Materials Research, Slovak Academy of Science, Watsonova 47, 040 01 Kosice, Slovakia; vhomolova@saske.sk; 4 Department of Structural Mechanics, Faculty of Civil Engineering, Technical University of Kosice, Vysokoskolska 4, 042 00 Kosice, Slovakia; ivo.demjan@tuke.sk

**Keywords:** HSLA steel, hot ductility, AlN precipitates, ductile fracture, brittle fracture

## Abstract

The article aims to investigate the effect of different austenitization temperatures on the hot ductility of C-Mn-Al High-Strength Low-Alloy (HSLA) steel. The thermo-mechanical simulator of physical processes Gleeble 1500D was used for steel hot ductility study. Hot ductility was estimated by measuring the reduction of area after static tensile testing carried out at temperatures in the range 600 °C to 1200 °C with the step of 50 °C. Evaluation of fracture surfaces after austenitization at 1250 °C and 1350 °C with a holding time of the 30 s showed significant differences in the character of the fracture as well as in the ductility. The fracture surfaces and the microstructure near the fracture surfaces of samples at a test temperature of 1000 °C for both austenitization temperatures were analyzed by Scanning Electron Microscopy (SEM), Light Optical Microscopy (LOM), and AZtec Feature analysis (particle analysis of SEM). AlN and AlN-MnS precipitates at grain boundaries detected by the detailed metallographic analysis were identified as the main causes of plasticity trough in the evaluated steel. Moreover, using Thermo-Calc software, it was found that AlN particles precipitate from solid solution below the temperature of 1425 °C.

## 1. Introduction

The techniques of continuous casting and direct rolling have been introduced and extended in order to increase process efficiency by saving on energy consumption, human labor, and production equipment. To ensure these processes are successful, there is a need to prevent the occurrence of surface defects, especially cracks, which occur quite often on the slab surfaces. In relation to the improvement of surface quality of continuously cast steels, it is important to study the hot ductility behavior of steels, considering their solidifying thermal history together with the crack formation during casting. Increasing the casting speed and strain rate during straightening can be one of the useful methods to decrease crack formation shortly after the casting zone [1,2,3,4,5,6].

Cracks are known to form in three distinct temperature ranges that depend on the ductility of steel: so-called high-temperature zone 1340 °C to solidus, intermediate-temperature range 800 °C to 1200 °C, and so-called low-temperature zone 700 °C to 900 °C [7].

The high-temperature zone (low strength and ductility) is generally believed to be due to the presence of liquid films in the interdendritic regions, which do not freeze until temperatures properly below the solidus are reached [8]. The liquid films contain a high level of sulfur, phosphorus, and other elements having a segregation coefficient less than unity. Loss of ductility in the intermediate-temperature zone is strongly dependent on the Mn/S ratio and the particular thermal history of the steel [7,8]. High Mn and Al concentrations in steel can significantly change the physicochemical conditions of non-metallic inclusion formation during steelmaking and casting processes [9]. The number of MnS inclusions in AHSS steels rises with Al content growth and decreases with N content growth. The amount of MnS is also affected by the stability of AlN in liquid steel and declines when AlN can form in liquid steel before solidification starts [10]. The increase in sulfur from 0.004 to 0.042 wt% was studied in a Fe-30%Mn-9%Al-1%Si-(0.9–1.2)%C-0.5%Mo steel (wt%). Results of this research show that controlled additions of S may promote soft and globular MnS that precipitates around AlN during solidification, thus reducing their negative effect [11]. Thermal history is considerably more complicated because it includes several variables such as melting/freezing in-situ, reheating from room temperature, cooling rate, isothermal holding temperature, and holding time [3].

Loss of ductility in the intermediate temperature zone generally decreases with decreased cooling rate, decreased holding temperature, and increased holding time. Strain rate does not appear to have much influence on the ductility [5,6,7,12].

The low-temperature zone (700 °C to 900 °C) is less known; however, there is a general agreement that it is usually associated with soluble aluminum in the steel and the precipitation of AlN at grain boundaries [7,13,14,15,16,17]. The strong nitride-forming element Al can form a huge amount of AlN inclusions in liquid steel. To control the formation of AlN inclusions based on the supersaturation of N and Al in liquid alloy steels, it is required to have correct thermodynamic data for the critical solubility limit of N and AlN in such steel grades during cooling and solidification [13]. For an explanation of AlN precipitation, it is also necessary to consider some physical mechanisms: for example, the precipitate volumetric misfit, the temperature-dependent Young’s modulus, configuration, temperature, and size-dependent interfacial energies, as well as the ratio between bulk and grain boundary diffusion [14]. The thermodynamic calculation processed by the authors of [15] shows that AlN is less stable than Al_2_O_3_ and FeAl_2_O_4_, and yet nitrides are frequently found in Fe-Al alloys. The authors further added that for AlN formation, there must be a region where the oxygen partial pressure is very low. If oxygen has been consumed in the process of forming oxides, the activity of oxygen in the alloy can become low enough to allow for the formation of nitrides [15]. The authors of [16] in their study provided as an example that when the aluminum content is 1.5 wt% in Fe-Mn-Al-N steel, the critical nitrogen contents for the AlN formation are 0.034, 0.02, and 0.01 wt% at 1548 °C, 1498 °C, and 1448 °C, respectively [16]. Manganese significantly increases the AlN solubility in liquid steel due to the large effect of manganese on nitrogen solubility [9]. In light-weight Fe-Mn-Al steels, it was detected that increasing the manganese content from 2 wt% to 20 wt% increases the total amount of AlN and MnS inclusions by 4–8 times [17]. The addition of titanium and vanadium was reported to be beneficial in preventing ductility decrease [18,19]. The others authors claimed titanium reduced ductility [20,21]. However, the optimal Ti/N ratio required to improve the ductility is still a discussed topic. The hypothesis of embrittlement of steel in the dual-phase region of austenite-ferrite was expressed by several authors [7,8,22,23]. As two main factors for embrittlement for this case, the presence of a thin proeutectoid (allotriomorphic) ferrite at the grain boundaries of austenite and the presence of tensile stresses in the slab surface shell were determined [22,23]. Dropping the C or Mn to low levels can promote ferrite formation, leading to very thin troughs, which make it considerably easier to avoid the problem of transverse cracking [19]. Due to the relatively low temperatures involved, the third low-ductility zone can be seen to be a factor only in the formation of surface and subsurface cracks [4,5,6].

Hot ductility is dependent on austenitic grain size and increases with decreasing grain size. However, in microalloyed steels, the dependence of hot ductility on grain size is less pronounced. The reason is the prevailing effect of precipitation of carbo-nitrides of alloying elements at grain boundaries [19]. Most research was focused on assessing the effect of different chemical compositions and different cooling rates on hot ductility, but the effect of different austenitization temperatures on hot ductility in microalloyed steels was studied only marginally.

This work is focused on finding the relation between austenitization temperature and hot ductility of the C-Mn-Al HSLA steel.

## 2. Materials and Methods

Experimental work was performed using the material concept of C-Mn-Al HSLA steel. The chemical composition of the steel is given in Table 1.

**Figure 1:**
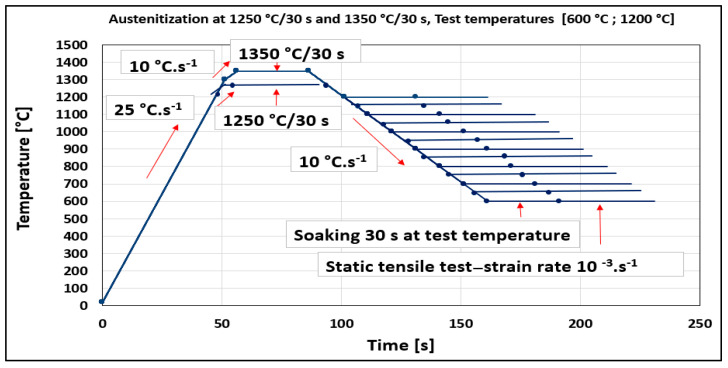
Heating and cooling cycle schedules are used for the determination of hot ductility.

The temperature range of plasticity trough was defined for the steel and two austenitization temperatures by Gleeble 1500D. Standard tensile test samples with nominal dimensions of ∅10 × 110 mm taken from transfer bar (i.e., semi-products during hot rolling between roughing and finishing train of hot strip mill) in the longitudinal direction to the direction rolling were used in the experiment. Samples were heated with heating rates of 25 °C·s^−1^ from ambient temperature to 1200 °C, followed with a heating rate of 10 °C·s^−1^ to 1250 °C. The same procedure was applied for austenitization temperature of 1350 °C (heating rate of 25 °C·s^−1^ to 1300 °C and 10 °C·s^−1^ to 1350 °C). Austenitization temperatures of 1250 °C and 1350 °C with a soaking time of 30 s were applied before cooling to the tensile test temperatures from 600 °C to 1200 °C by cooling rate of 10 °C·s^−1^ followed by soaking at the temperature for the 30 s. A deformation rate of 10^−3^·s^−1^ was used for all test temperatures. Per cent of reduction of area (RA) was used to evaluate plasticity. RA was obtained as RA = (A_0_ − A_f_) × 100/A_0_, where A_0_ and A_f_ are the initial cross-sectional area and the final cross-sectional area (after rupture) of the tensile sample, respectively, and was given in %. The experimental schedule for the investigation of thermal cycles and ductility is given in Figure 1.

To investigate microstructure fracture surfaces and the chemistry of phases, Light Optical Microscopy (LOM), Scanning Electron Microscopy (SEM), X-ray analysis (EDX), and SEM-based AZtec Feature analysis were used. Feature analysis is automated SEM-based area scanning focused on positioning and identification of particles and features, such as nonmetallic inclusions. The morphological and chemical data of the features were evaluated. The longitudinally cut samples were mounted in conductive resin. The surfaces of samples were metallographically prepared and etched with 3% Nital. The area of 5 × 5 mm was scanned closed to the fracture line. The size of smallest identified feature was set to 0.86 μm. The chemical composition of the C-Mn-Al HSLA steel was determined using a OBLF QSG 750 analyzer (OBLF, Witten, DE, Germany) by Optical Emission Spectral Analysis. The thermodynamic calculations were performed using Thermo-Calc software (version: TCC S-version) with commercial thermodynamic database TCFE6 [24]. Phase equilibria were calculated for the temperature range 400–1600 °C.

## 3. Results

**Figure 2:**
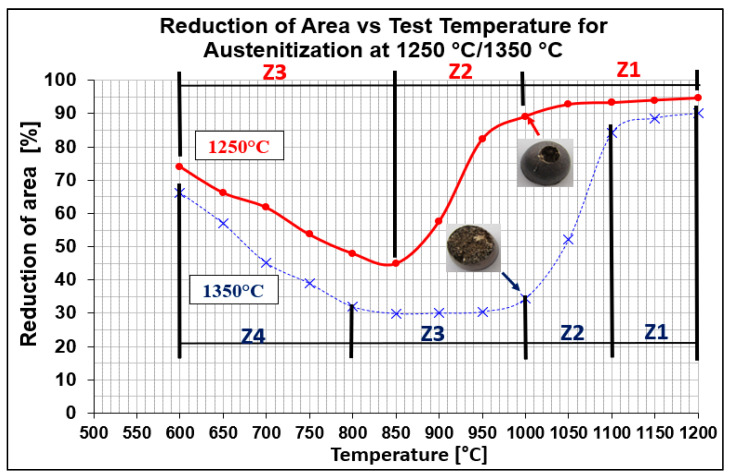
Reduction of area (RA) vs. test temperature for austenitization at 1250 °C/30 s and 1350 °C/30 s, red and blue lines, respectively.

Hot ductility was evaluated by measuring the reduction of area (RA) after static tensile testing at temperatures from 600 °C to 1200 °C with 50 °C steps. Two austenitization temperatures of 1250 °C/30 s and 1350 °C/30 s were applied. The results are plotted in Figure 2. As can be seen from Figure 2, RA values of samples austenitized at 1250 °C (red line) were higher compared to samples austenitized at 1350 °C (blue line). Whereas austenitization at 1350 °C resulted in a decrease in plasticity to 30% at a temperature range of 950 °C to 850 °C, austenitization at 1250 °C resulted in a decrease in plasticity to 45% at 850 °C. The most significant difference in ductility was observed at the test temperature of 1000 °C.

Evaluation of RA for both austenitization temperatures depends on testing temperatures, which showed that curves had two local minima. The whole interval of test temperatures was divided as followed:

- austenitization temperature 1350 °C was described by four temperature zones (Z1–Z4);
- austenitization temperature 1250 °C was characterized only by three temperature zones (Z1–Z3).

**Figure 3:**
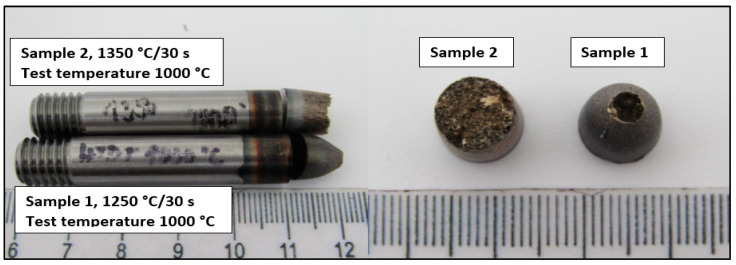
Macro-view of samples after hot ductility test.

The macroscopic view of samples tested by a static tensile test at temperature 1000 °C is shown in Figure 3.

From macroscopic observation, it is evident that high ductility was observed on the sample tested at 1250 °C/30 s compared to the sample tested at 1350 °C/30 s. The ductility of Sample 2 is characterized as limited with the brittle fracture.

**Figure 4:**
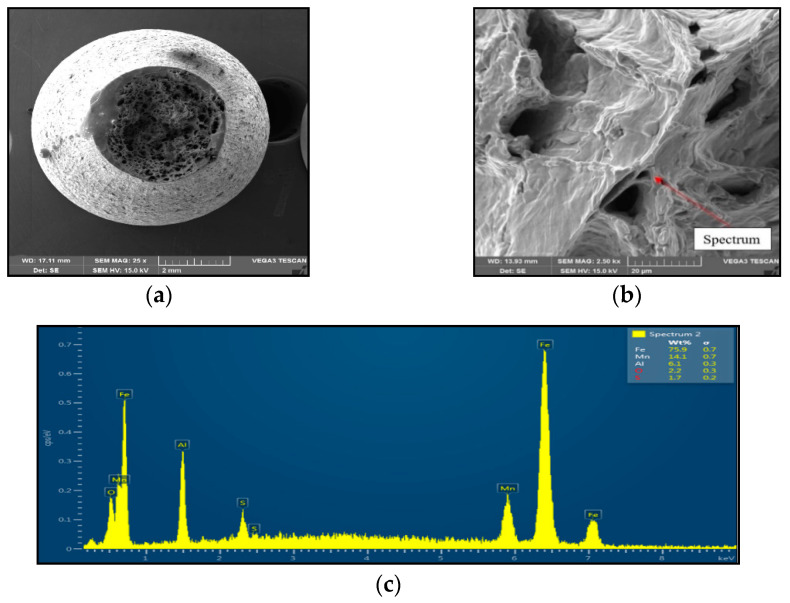
Ductile dimpling after austenitization at 1250 °C/30 s and test temperature of 1000 °C, SEM. (**a**) 3000×-magnification; (**b**) 2500×-magnification; (**c**) EDX spectrum.

**Figure 5:**
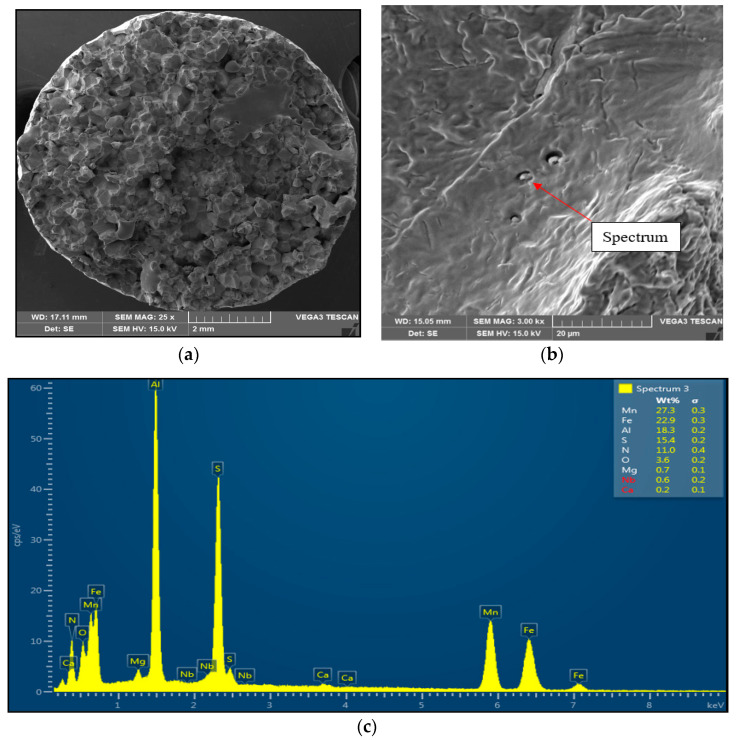
Intergranular brittle fracture surface after austenitization at 1350 °C/30 s and test temperature of 1000 °C, SEM. (**a**) 25× magnification; (**b**) 3000× magnification; (**c**) EDX spectrum of coarse AlN-MnS precipitate.

The detailed observations of fracture surfaces of Sample 1 and Sample 2 are illustrated in Figure 4 and Figure 5, respectively.

Sample 1 (1250 °C/30 s) showed significant stretching and formation of the neck, and finally at the center of the neck the fracture formed, as is shown in Figure 4. The fracture mechanism represents the occurrence of dimples, which grow and coalesce up to fast shear fracture with the formation of cup and cone fracture surface. Dimple fracture indicates ductile high-energy break with a relative good reduction in area. Dimples were distributed evenly, with the random distribution of dimple sizes (about 20 μm to 200 μm) oriented longitudinally to the sample axis, with the exception of the fast fracture, where dimples are oriented in the shear direction. EDX spectrum analysis from the surface of the dimples showed the presence of 6.1 wt% Al and 14.1 wt% Mn; the nitrogen was under the detection limit.

Sample 2 (1350 °C/30 s) showed mild stretching and almost no neck formation with a small reduction in area, as is shown in Figure 5. The fracture was brittle and intergranular with low energy to break, caused probably by coarsening the former austenite grain and second-phase particles precipitating at grain boundaries. The presence of coarse particles of second phase at grain boundaries was confirmed at the 3000× magnification. The particles were distributed along grain boundaries, their shape was plate-like, and their size was about 1 μm to 2 μm. The EDX analysis spectrum of particle showed the presence of 27.3 wt% Mn, 18.3 wt% Al, 15.4 wt% S, and 11.0 wt% N. Based on the chemical composition of results, it can be assumed that the particles of the second phase found at grain boundaries were coarse AlN-MnS precipitates.

**Figure 6:**
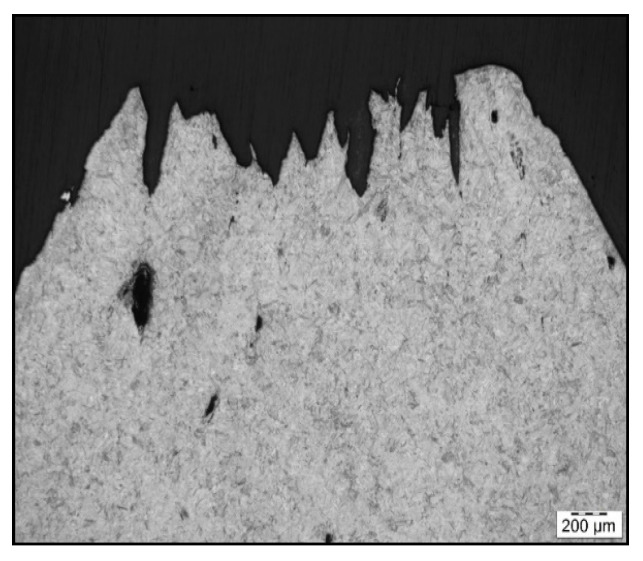
Fracture line after austenitization at 1250 °C/30 s and testing at 1000 °C, LOM.

**Figure 7:**
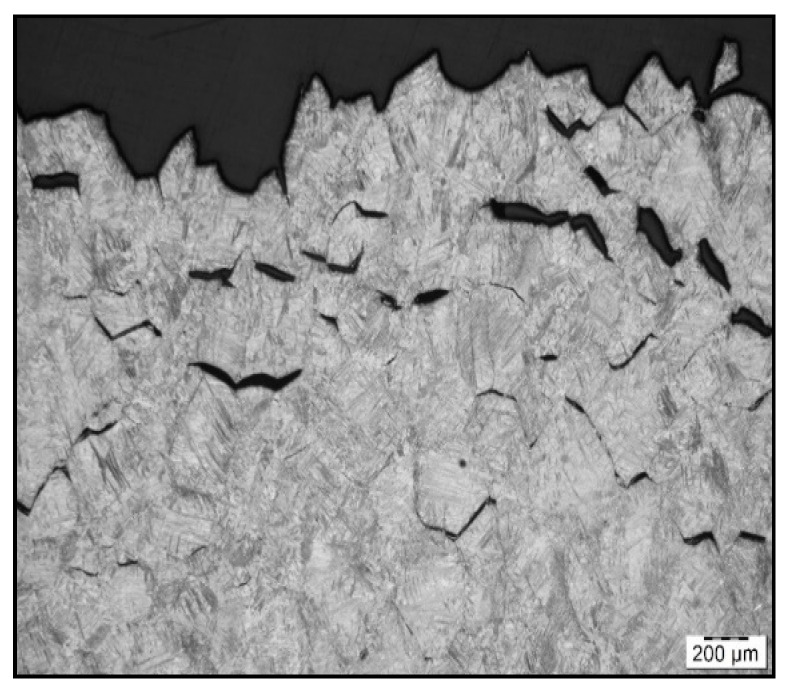
Fracture line after austenitization at 1350 °C/30 s, and testing at 1000 °C, LOM.

To confirm grain coarsening and presence of second-phase particles at grain boundaries LOM of the cross-section at fracture line of samples after austenitization temperature of 1250 °C/30 s and 1350 °C/30 s was used. The appearance of the fracture line (Figure 6 and Figure 7) and microstructures below them were quite different, with apparent fracturing at coarse austenite boundaries in Figure 7.

**Figure 8:**
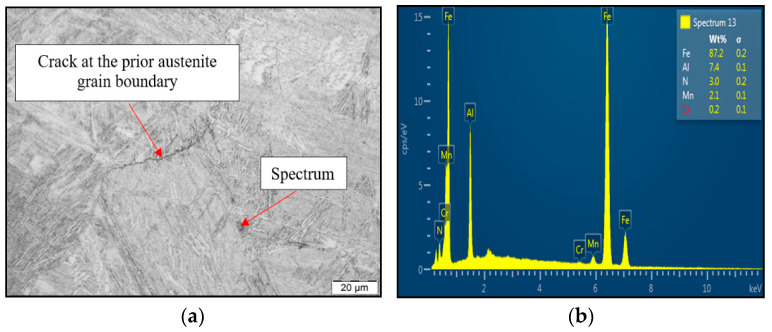
(**a**) LOM of cross-section close to fracture line; (**b**) EDX spectrum of found coarse AlN precipitate.

To reveal the reason for susceptibility to ductility decrease and tendency to brittle fracture formation if the material was exposed to austenitization temperature of 1350 °C/30 s, SEM and EDX analysis have been used, as shown in Figure 8. LOM of the cross-section at fracture line of samples after austenitization temperature of 1350 °C/30 s confirmed the presence of the second phase excluded at the grain boundaries. Based on chemical composition from EDX analysis, it can be stated that the particle shown in Figure 8a was coarse AlN precipitate. The size of the AlN precipitate is about 4 μm, and the shape was plate-like. Based on the results, AlN precipitation at grain boundaries has been detected to be the reason for material low ductility.

**Figure 9:**
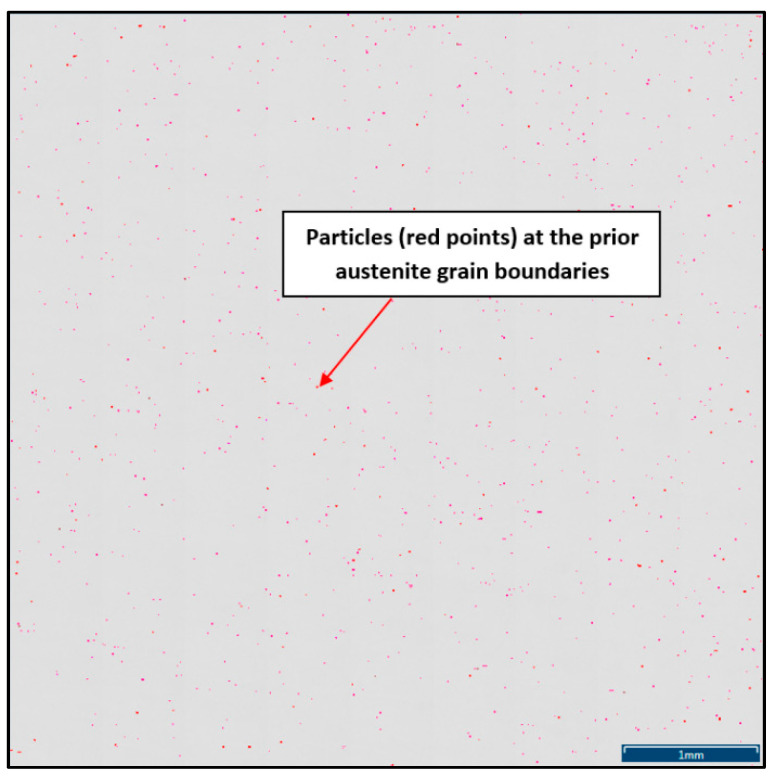
Distribution of non-metallic particles at the former austenite grain boundaries identified on the total area of 5 × 5 mm positioned and evaluated by SEM AZtec Feature analysis.

To define the ratio between AlN, AlN-MnS, and the other particles from the Al+Mn subclass, SEM-based AZtec Feature analysis on an area of 5 × 5 mm was performed. The distribution of non-metallic particles at the former austenite grain boundaries is shown in Figure 9. The analysis results showed a significant predominance of AlN particles in comparison with other found AlN-MnS, AlMgO, AlN-AlO, and AlO in the ratio 586:203:152:60:2, respectively.

**Figure 10:**
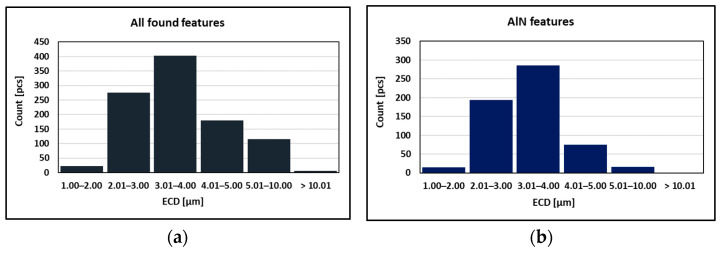

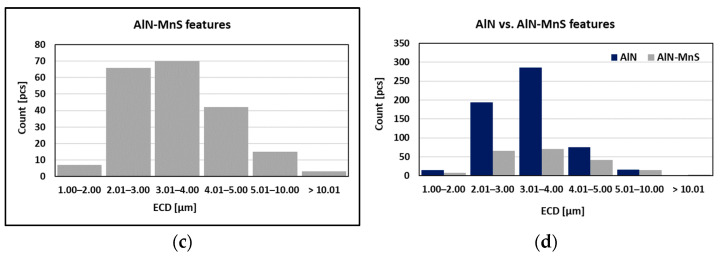
Found features: (**a**) All; (**b**) AlN; (**c**)AlN-MnS; (**d**) AlN vs. AlN-MnS.

As can be seen in Table 2 and Figure 10, the particles with sizes within 3.01–4.00 μm represents the greatest volume, followed by particles with sizes within 2.01–3.00 μm, 4.01–5.00 μm, 5.01–10.00 μm, and 1.00–2.00 μm and greater than 10 μm. The size of smallest identified feature was set to 0.86 μm.

**Figure 11:**
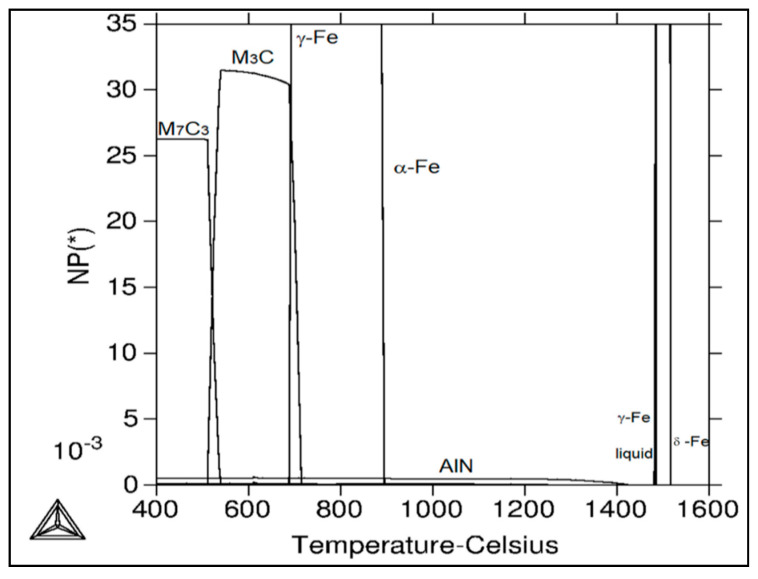
Calculated mole fraction of stable phases in the dependence of temperature for the C-Mn-Al HSLA steel using Thermo—Calc software.

Table 3 and Figure 11 show calculated mole fractions of stable phases’ independence of temperature for the experimental material using Thermo-Calc software. At the temperature of 1517 °C and above, only the liquid phase exists. The following phases are present in the given temperature ranges: from 1517 °C to 1485 °C, a two phase region with δ-ferrite and liquid; from 1485 °C to 1484 °C; δ–ferrite, austenite, and liquid; from 1484 °C to 1480 °C, austenite and liquid; from 1480 °C to 1425 °C, austenite; from 1425 °C to 1278 °C, AlN and austenite; from 1278 °C to 957 °C, AlN, austenite, and Ti-nitride; from 957 °C to 894 °C, AlN, austenite, Ti-nitride, and NbTi-carbide; from 894 °C to 715 °C, AlN, austenite, ferrite, Ti-carbide/nitride, and NbTi-carbide; from 715 °C to 688 °C, AlN, austenite, ferrite, Ti-carbide/nitride, NbTi-carbide, and cementite; from 688 °C to 539 °C, AlN, ferrite, Ti-carbide/nitride, NbTi-carbide, and cementite; from 539 °C to 524 °C, AlN, ferrite, Cr-carbide *, Ti-carbide/nitride, NbTi-carbide, and cementite; from 524 °C to 511 °C, AlN, ferrite, Cr-carbide *, Ti-carbide/nitride, V-carbide, NbTi-carbide, and cementite; and from 511 °C to 400 °C, AlN, ferrite, Cr-carbide *, Ti-carbide/nitride, -carbide, and NbTi-carbide. According to the calculations, AlN starts to precipitate from austenite solid solution at 1425 °C. Its amount is relatively stable up to lower temperatures. In addition to AlN, cementite (M_3_C) and Cr-carbide * (M_7_C_3_) are stable phases at lower temperatures of the investigated temperature range. MX phases (Ti-carbide/nitride, V-carbide, NbTi-carbide) were calculated as equilibrium phases (see Table 2); however, their amounts are very low.

## 4. Discussion

This work is focused on finding the relation between austenitization temperature and hot ductility of C-Mn-Al HSLA steel. Hot ductility results defined by the reduction of area for particular test temperatures from 600 °C to 1200 °C are shown in Figure 2. From graphical dependences, it is evident that the austenitization temperature influenced the shift of the temperature range of plasticity drop.

To better explain the causes of the decrease in plasticity, the individual austenitization temperatures were divided into main zones. For the austenitization at 1250 °C/30 s, there are three zones: zone 1 in the temperature range of 1200–1000 °C with plasticity 95–90%, zone 2 in the temperature range of 1000–850 °C with a significant decrease in plasticity from 85% to 45%, and zone 3 in the temperature range of 850–600 °C with an increase in plasticity from 45% to 75%. For the austenitization at 1350 °C/30 s, there are four zones: zone 1 in the temperature range of 1200–1100 °C with plasticity 90–85%, zone 2 in the temperature range of 1100–1000 °C with a significant decrease in plasticity from 85% to 35%, zone 3 in the temperature range of 1000–800 °C with low plasticity from 35% to 30%, and zone 4 in the temperature range of 800–600 °C with an increase in plasticity from 35% to 65%.

Plasticity in the temperature range of 1200–1000 °C (which is characterized by zone 1 for both austenitization temperatures) is very sensitive to the amounts of impurities, such as sulfur and oxygen, in the steel. Thus, the high plasticity values can be attributed to the favorable Mn/S ratio as well as the small amount of non-coarse precipitates, in particular AlN, formed at the austenitic grain boundaries [7,18].

The decrease in plasticity in the range of 1100–800 °C, which in this case is characterized by zone 2 at austenitization of 1250 °C/30 s and zones 2 and 3 at austenitization of 1350 °C/30 s, is often attributed to the unfavorable Mn/S ratio and particular thermal history of the steel. Steel with Mn/S ratio above 60 is not embrittled, because the sulfur is tied to the stable phase, MnS, which precipitates in the matrix, not predominantly at the grain boundaries like FeS. Thermal history is significantly more complex, because it includes several variables such as melting/freezing in-situ, reheating from room temperature, cooling rate, isothermal holding temperature, and holding time. Loss of ductility in this temperature range usually decreases with decreased cooling rate, decreased holding temperature and increased holding time. Strain rate does not appear to have much effect on the ductility [7,18,20]. In the steel we studied, a significant decrease in plasticity was associated with the weakening of the austenitic grain boundaries, which was caused by coarse precipitates, especially AlN and AlN MnS. This fact was confirmed by the results of metallographic analyzes performed on samples at a test temperature of 1000 °C (for both austenitization temperatures), where the largest difference in plasticity was measured, up to 55%. Analysis of the fracture surfaces (Figure 5 and Figure 6) showed significant differences in the fracture mechanism. While Sample 1 (austenitization 1250 °C/30 s) showed significant stretching and formation of the neck, and finally at the center of the neck the fracture formed, Sample 2 (austenitization 1350 °C/30 s) showed mild stretching and almost no neck formation, with a small reduction in area. The fracture mechanism at Sample 1 was the occurrence of dimples, while fracture at Sample 2 was brittle and intergranular with low energy to break. The reason for the brittle fracture in Sample 2 was further investigated. As it can be seen in Figure 6 and Figure 9, the coarse precipitates of AlN and AlN-MnS at the grain boundaries were detected on the fracture surfaces as well as in the cross-section close to the fracture line. To define the ratio between AlN, AlN-MnS, and the other particles from the Al+Mn subclass, SEM-based AZtec Feature analysis on an area of 5 × 5 mm was performed. The analysis results show a significant predominance of AlN particles in comparison with other found AlN-MnS, AlMgO, AlN-AlO, and AlO in the ratio 586:203:152:60:2, respectively. As can be seen in Table 2 and Figure 11, the particles with sizes within 3.01–4.00 μm represents the biggest volume, followed by particles with size within 2.01–3.00 μm, 4.01–5.00 μm, 5.01–10.00 μm, and 1.00–2.00 μm and greater than 10 μm. Considering several types of research on fracture surfaces, there is clear evidence that poor hot ductility of investigated steels was associated with the presence of AlN and/or AlN-MnS particles [10,11,17,25,26,27,28,29,30,31,32,33]. Precipitation kinetics simulations performed by the authors of [29] showed that longer holding time leads to growth of AlN at grain boundaries, a coarsening of MnS at dislocations, and a coarsening and growth of AlN at MnS. The authors of [31] found that in FeCrAl steel, the number of AlN particles increases with increasing cooling rate, although the volume fraction is relatively unaffected. The authors of [10] found that an increase in Al content from 0.5% to 6% increased the number of complex MnS inclusions by approx. 4 times. Based on results in Figure 11 and Table 3, the AlN particles are present in microstructure from 1425 °C to a temperature of 400 °C. However, the AlN precipitates are stable below 400 °C as well. Based on Thermo-Calc software results, it can be stated that at a temperature higher than 1200 °C, small AlN particles dissolve, and thermodynamically more stable particles at grain boundaries grow. At a temperature higher than 1425 °C AlN, particles dissolve fully in γ-Fe. It can be stated that experimental results correspond with those calculated using Thermo-Calc software quite well. The results obtained by the authors of [13] showed that the solubility of AlN ranged from 1449 °C to 1549 °C in TWIP multi-component Fe-Mn-Si-C-Al steel (24 wt% Mn, 0.3 wt% Si, 0.6 wt%C, 1.3 wt% Al). Some authors used FactSage software to calculate the solubility of AlN in various chemical steel concepts [10,17,33]. The authors of [17] determined that AlN do not precipitate in liquid steel with the composition of 0.12 wt% C, 2.03 wt% Mn, 3 wt% Al, 3 wt% Si, and 0.0004 wt% N or 0.12 wt% C, 5.1 wt% Mn, 2.8 wt% Al, 3 wt% Si, and 0.0006 wt% N. On the other hand, in steel with a composition of 0.12 wt% C, 20.6 wt% Mn, 2.8 wt% Al, 3.5 wt% Si, and 0.0011 wt% N, AlN can be formed in liquid steel during cooling from 1599 °C. The authors of [10] investigated the solubility of AlN in 7 different chemical concepts of AHSS steels, but none of them corresponded very well to our C-Mn-Al HSLA steel. For steel with an Al content of 0.9 wt%, the stability of AlN was determined at 1370 °C. The authors of [33] calculated that in steel with 0.04 wt% C, 0.24 wt% Mn, 3.0 wt% Si, 0.01 wt% Al, and 0.004 wt% O, AlN is formed at the last stage of solidification, and the condition of its formation is low oxygen content in the steel.

The decrease in plasticity in the temperature range of 850–600 °C, which in this case is characterized by zone 3 (austenitization of 1250 °C/30 s) and zone 4 (austenitization of 1350 °C/30 s), is often attributed to a two-phase (austenite-ferrite) region. Controlling factors for this embrittlement are grain boundary sliding and the localization of strain to the proeutectoid ferrite film along the austenite grain boundary, which is produced by the γ→α transformation. Although much research has been done, the embrittlement mechanism has not been understood completely yet [4,5,6,7]. Based on the results of previous research [34] where the γ→α phase transformation temperatures Ar_3_-Ar_1_ (894–688 °C) were determined by Thermo-Calc software for C-Mn-Al HSLA steel, it can be believed that the above-mentioned mechanism was the cause of embrittlement in zone 3 (austenitization of 1250 °C/30 s) and zone 4 (austenitization of 1350 °C/30 s).

Some studies declare that the critical RA value for surface crack formation during the continuous casting process is less than 60% [1,25]. Based on these findings, RA less than 60% was measured in the range of 700–900 °C at austenitization of 1250 °C/30 s and in range of 630–1080 °C at austenitization of 1350 °C/30 s. Despite a wide range of hot ductility studies performed for different steel concepts, none of them corresponds very well to investigated C-Mn-Al HSLA steel chemistry. The results of this research, therefore, extend knowledge related to hot ductility, especially for HSLA steels.

## 5. Conclusions

In the present study, the effect of austenitization temperature at 1250 °C and 1350 °C with 30 s soaking on the hot ductility of C-Mn-Al HSLA steel was studied. Based on results achieved, the following conclusions can be stated:

- For the test temperature of 1200 °C, maximum plasticity of 90% and 85% for austenitization temperatures 1250 °C/30 s and 1350 °C/30 s, respectively, was measured. Minimum plasticity of 45% for test temperature of 850 °C and 30% for test temperatures from 850 °C to 950 °C and austenitization temperatures of 1250 °C/30 s and 1350 °C/30 s, respectively, was determined.
- While the sample after austenitization at 1250 °C/30 s showed a ductile fracture, the sample after austenitization at 1350 °C/30 s showed a small reduction of area, with low-energy brittle intergranular fracture.
- AlN and AlN-MnS coarse precipitates at austenite grain boundaries are supposed to be the reason for brittle intergranular fracturing.
- Using Thermo-Calc software, it was found that AlN particles precipitate from solid solution below the temperature of 1425 °C.

## Figures and Tables

**Table 1:** Chemical composition of the C-Mn-Al HSLA steel.

Component	C	Mn	Si	P	Al	Cr + Mo	Nb + Ti
wt%	max. 0.18	max. 2.1	max. 0.14	max. 0.04	max. 0.7	max. 1.0	max. 0.15

**Table 2:** Morphological and chemical characteristic of Features—ECD—equivalent circular diameter.

Composition of Particles	Number of Features	ECD [μm] 1.00–2.00	ECD [μm] 2.01–3.00	ECD [μm] 3.01–4.00	ECD [μm] 4.01–5.00	ECD [μm] 5.01–10.00	ECD [μm] > 10.01
AlN	586	15	194	286	75	16	0
AlN-MnS	203	7	66	70	42	15	3
AlMgO	152	0	8	25	41	75	3
AlN-AlO	60	0	8	22	21	9	0
AlO	2	0	0	0	1	1	0
Total	1003	22	276	403	180	116	6

**Table 3:** Calculated phase equilibria in temperature range 400–1600 °C.

Phase Equilibria	T [°C]
AlN, ferrite (α), M_7_C_3_ *, MX1(TiX), MX2(VC), MX3((NbTi)C)	400–511
AlN, ferrite (α), M_7_C_3_ *, MX1(TiX), MX2(VC), MX3((NbTi)C), cementite	511–524
AlN, ferrite (α), M_7_C_3_ *, MX1(TiX), MX3((NbTi)C), cementite	524–539
AlN, ferrite (α), MX1(TiX), MX3((NbTi)C), cementite	539–688
AlN, ferrite (α), austenite (γ), MX2(TiX), MX3((NbTi)C), cementite	688–715
AlN, ferrite (α), austenite (γ), MX2(TiX), MX3((NbTi)C)	715–894
AlN, austenite (γ), MX2(TiN), MX3((NbTi)C)	894–957
AlN, austenite (γ), MX2(TiN)	957–1278
AlN, austenite (γ)	1278–1425
austenite (γ)	1425–1480
Liquid, austenite (γ)	1480–1484
Liquid, δ-ferrite, austenite (γ)	1484–1485
Liquid, δ-ferrite	1485–1517
Liquid	1517-

* Relating to the chemical composition of the experimental steel, the M_7_C_3_ carbide can be considered to be Cr-carbide.

## Data Availability

Data sharing is not applicable to this article.
